# Disinhibition of Human Primary Somatosensory Cortex After Median Nerve Transection and Reinnervation

**DOI:** 10.3389/fnhum.2020.00166

**Published:** 2020-05-15

**Authors:** Per F. Nordmark, Roland S. Johansson

**Affiliations:** ^1^Department of Integrative Medical Biology, Physiology Section, Umeå University, Umeå, Sweden; ^2^Umeå Center for Functional Brain Imaging, Umeå University, Umeå, Sweden; ^3^Department of Surgical and Perioperative Sciences, Section for Hand and Plastic Surgery, Umeå University, Umeå, Sweden

**Keywords:** humans, hand, peripheral nerve injury, touch, magnetic resonance imaging, somatosensory cortex, cortical plasticity

## Abstract

Despite state-of-the-art surgical and postoperative treatment, median nerve transection causes lasting impaired hand function due to limitations in the nerve’s reinnervation ability. The defective innervation and thus controllability of the affected hand can shape the brain’s control of manual behaviors. Earlier studies of changes in the processing of tactile stimuli have focused mainly on stimulation of the reinnervated hand and lack sufficient control over the brain’s use of the tactile input in perceptual terms. Here we used fMRI to measure brain activity (BOLD-signal) in 11 people with median nerve injury and healthy controls (*N* = 11) when performing demanding tactile tasks using the tip of either the index or little finger of either hand. For the nerve-injured group, the left median nerve had been traumatically transected in the distal forearm and surgically repaired on average 8 years before the study. The hand representation of their contralesional (right) primary somatosensory cortex (S1) showed greater activity compared to controls when the left reinnervated index finger was used, but also when the left-hand little finger and the fingers of the right hand innervated by uninjured nerves were used. We argue that the overall increase in activity reflects a general disinhibition of contralesional S1 consistent with an augmented functional reorganizational plasticity being an ongoing feature of chronic recovery from nerve injury. Also, the nerve-injured showed increased activity within three prefrontal cortical areas implicated in higher-level behavioral processing (dorsal anterior cingulate cortex, left ventrolateral prefrontal and right dorsolateral prefrontal cortex), suggesting that processes supporting decision-making and response-selection were computationally more demanding due to the compromised tactile sensibility.

## Introduction

Despite state-of-the-art surgical repair and post-operative rehabilitation, traumatic transection of the median nerve at the distal forearm level usually results in a lasting impairment of hand function due to defective sensory innervation of the three radial digits and of intrinsic hand muscles (Jaquet et al., 2001; Lundborg and Rosén, 2007; Chemnitz et al., 2013; Pederson, 2014). Most of the attainable peripheral reinnervation is considered to be reached about 2 years after surgical repair of such an injury (Seddon et al., 1943; Sunderland, 1947; Buchthal and Kühl, 1979; Burnett and Zager, 2004; Grinsell and Keating, 2014). Residual innervation deficiencies involve loss of primary sensory and motor neurons, misdirected reinnervation of axons concerning location and type of end-organs, and abnormal conduction and stimulus coding properties of reinnervated neurons (Buchthal and Kühl, 1979; Hallin et al., 1981; Mackel et al., 1983; Liss et al., 1996; Allodi et al., 2012; de Ruiter et al., 2014; Krarup et al., 2017).

The defective reinnervation might affect brain processes that normally support manual behaviors. Animal studies have shown that peripheral nerve lesions can cause substantial functional reorganizations in both the contralesional primary somatosensory area (S1; Paul et al., 1972; Wall et al., 1986; Merzenich and Jenkins, 1993; Florence et al., 1994) and the primary motor area (M1; Sanes et al., 1990; Kaas, 1991; Nudo et al., 1997; Chen et al., 2002). However, they also indicate that a transected median nerve after repair and reinnervation largely resumes its original cortical projection territory in S1, but distorted internal topography within the nerve projection area can be present (Wall et al., 1986; Merzenich and Jenkins, 1993; Florence et al., 1994). In humans, previous functional magnetic resonance imaging (fMRI) studies have reported that tactile stimulation of digits can activate the contralateral S1 to a greater extent after median nerve injury and reinnervation than in healthy individuals (Hansson and Brismar, 2003; Taylor et al., 2009; Fornander et al., 2010; Chemnitz et al., 2015). In addition, structural changes have been reported in gray and white matter mainly within brain areas involved in higher-level planning and control of hand actions (Taylor et al., 2009; Nordmark et al., 2018) and have been interpreted as reflecting changes in neural processing linked to restrictions in the natural dexterity repertoire and to processes helping functional compensation of somatosensory deficiencies of the hand (Nordmark et al., 2018).

Using fMRI, here we addressed some of the many remaining issues of long-term changes in brain processing by examining 11 people who had suffered a median nerve transection in their left distal forearm followed by surgical repair at least 2.5 years earlier (mean = 8 years). Previous studies of altered brain responses to tactile stimuli after unilateral median nerve injury and reinnervation have focused mainly on stimulations within the reinnervated skin area (Hansson and Brismar, 2003; Taylor et al., 2009; Fornander et al., 2010; Chemnitz et al., 2015). However, injury related changes in cortical responses to tactile input from non-median innervated skin areas within the affected hand can be expected since the activity within S1’s finger representations dynamically interact under normal conditions (Ruben et al., 2006; Lipton et al., 2010; Reed et al., 2010; Thakur et al., 2012; Martuzzi et al., 2014). Likewise, effect of injury on responses to inputs from the hand of the non-injured arm can be expected since stimulation of one hand can not only give rise to contralateral cortical responses, but also to ipsilateral activity changes in healthy individuals, including within S1 (Hlushchuk and Hari, 2006; Klingner et al., 2015; Tamè et al., 2016; Tal et al., 2017). We tested these propositions by tactile stimulation targeting the tip of the index finger innervated by the reinnervated median nerve, but also the tip of the little finger of the same hand innervated by the uninjured ulnar nerve and the tips of the index and little fingers of the other unaffected hand. Furthermore, previous imaging studies on changes in brain processing after median nerve reinnervation rarely controlled adequately for the task/attentional set (Sakai, 2008), although it is well established that the neural processing of tactile stimuli varies with task requirements even in early somatosensory areas (Johansen-Berg et al., 2000; Staines et al., 2002; Nelson et al., 2004; Nordmark et al., 2012; Gomez-Ramirez et al., 2016; Puckett et al., 2017). Two tactile tasks were performed in the scanner in this study, both of which required the participants to continuously attend to and process the peripheral tactile afferent signals during the entire task periods.

In the tactile threshold-tracking task participants tracked continuously their perceptual tactile thresholds at 20 Hz sinusoidal skin indentations (Nordmark et al., 2012), which implied that the effective stimulus intensity was approximately equal across all tested fingers of all participants, including reinnervated index fingers. Equalizing the effective stimulus intensity enabled consistent comparisons across experimental conditions by minimizing possible effects of stimulus intensity on evoked blood-oxygen-level-dependent (BOLD) activity (Arthurs et al., 2000; Nelson et al., 2004; Siedentopf et al., 2008).

In the *tactile* *oddball-detection task*, the participant’s assignment was to detect rarely occurring epochs of 40 Hz sinusoidal stimulations among regular epochs of 20 Hz stimulations. Suprathreshold stimulation intensities were used and the intensity was for each stimulated finger standardized to a multiple of the perceptual threshold, again with the aim of equating the effective stimulus intensity across all stimulated fingers. Our rationale for including the oddball-detection task was twofold. First, various brain areas, including S1, may be more critically involved in tasks requiring tactile frequency discrimination than in tasks requiring detection of stimuli at threshold (LaMotte and Mountcastle, 1979; Romo et al., 2012). Second, a nonlinear system, normal or degraded, can be meaningfully characterized only by studying its output in response to a variety of inputs. In this respect, the suprathreshold stimulation amplitudes in the oddball task complemented the near-threshold amplitudes used during the tactile threshold-tracking task.

The participants also performed a *visual threshold-tracking task* while receiving task-irrelevant tactile stimulation of the fingers. We included this task to investigate whether the nerve-injured participants maintained the ability of healthy individuals to suppress tactile afferent-induced activity in somatosensory brain areas during tasks primarily controlled by sensory information in other modalities, in this case the visual modality (Hsiao et al., 1993; Johansen-Berg et al., 2000; Nordmark et al., 2012).

Although previous research gives rise to expectation of effects of median nerve injury in S1, there are reasons to believe that neural processing in areas beyond S1 might be affected as well (Taylor et al., 2009; Nordmark et al., 2018). Therefore, we first searched the whole brain for effects of median nerve injury on blood-oxygen-level dependent (BOLD) activity, and then performed regional analyses focused on areas where such effects were discovered, which indeed included contralesional S1. In short, our results indicate that median nerve injury after reinnervation was associated with increased activity in finger representations in the contralesional S1 during the tactile tasks irrespective of finger used, i.e., when the left reinnervated finger was used as well as when the left little finger and the right-hand fingers innervated by intact nerves were used. Similarly, certain prefrontal cortical areas implicated in decision-making and response selection showed elevated brain activity during the tactile tasks regardless of finger used. During the visual task, we found no effect on the brain activity of the nerve-injured participants, indicating that their brains could normally suppress task-irrelevant tactile afferent signals and that the increased activity during the tactile task was task-modality specific.

## Materials and Methods

### Study Participants

Our study participants consisted of 11 median nerve-injured right-handed adults (three females, eight males) and 11 healthy persons matched to age, gender and handedness. All gave their written informed consent in accordance with the Declaration of Helsinki and the ethics committee of Umeå University approved the study (DNR 07-087M).

All injured participants suffered from a complete median nerve transection in their left distal forearm proximal to the wrist caused by sharp trauma. The distance from the tip of the index finger to the transection site ranged between 18 and 27 cm (mean = 20 cm). Within 24 h of injury, all had undergone surgery with primary epineural suture of the nerve at the Department of Hand Surgery, University Hospital of Umeå. None of the participants suffered from diabetes, severe pain from the hands or any diagnosed neurological disorders.

At the time of the study, the mean age of the nerve-injured participants was 43.0 years (SD = 15.0, range 18–64). On average, the injury had taken place 8.1 years earlier (SD = 4.5, range 2.5–17.1). The nerve-injured participants were the same people who made up the group of left-side injured in a previous study of structural changes in the brain following median nerve injury (Nordmark et al., 2018). That study offers more detailed information about the characteristics of the nerve-injured participants in standard clinical terms and on their responses to “Cold Intolerance Symptom Severity” (CISS) questionnaire and the “Disabilities of the Arm, Shoulder and Hand” (DASH) questionnaire, as well as a description of how the injury affected their everyday life. In short, the 11 nerve-injured participants appeared quite homogeneous regarding the clinical outcome of the injury. Although, none had constraints in active range of motion they all showed a modest thenar muscle atrophy of the affected hand. Injuries to tendons had occurred in all participants and injuries to arteries in a few. When the nerve was sutured also these injuries were surgically repaired. In agreement with previous reports on corresponding injuries, all nerve-injured had impaired static 2-point discrimination threshold (>7 mm) at the tips of reinnervated digits (Rosén, 1996). The mean age difference between each nerve-injured and gender matched control person was 0.3 years (SD = 2.0, range 4.0 to −3.2). The members of the control group were recruited through local posters and candidates were excluded if they had any problems with hand functions.

### General Procedure and Apparatus

The participants lay supine in a 3 T General Electric scanner with a 32-channel head coil (GE Medical Systems, Milwaukee, WI, USA). The head was stabilized with sponges to the head-coil and soundproof earphones combined with earplugs reduced scanner noise. Cushions supported the arms down to the wrist. Mirrors attached to the head coil allowed the participant to view a computer screen located in the rostral end of the bore.

**Figure 1:**
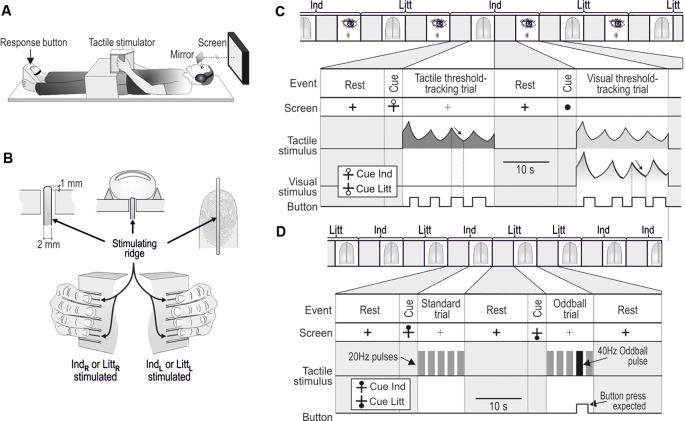
Apparatus and tasks in the MR scanner. **(A)** With either the left of the right hand, the participants grasped the box-shaped tactile stimulator anchored to a wooden frame extending above their hips. The big toe of the contralateral foot operated the response button. The participants could view a computer screen *via* mirrors. **(B)** Either the tip of the index (Ind) or the little (Litt) finger was stimulated *via* perpendicular movements of ridges protruding above the surface of the box. Ind_L_, Litt_L_,and Ind_R_,Litt_R_ refer to left- and right-hand fingers, respectively. **(C)** One of 24 paired tactile and visual threshold-tracking trials consecutively performed during a scanning session involving unilateral index (Ind) and little (Litt) finger stimulation. The stimulus in the tactile trial (represented as sinusoidal RMS value) was replayed during the following visual trial. Participants pressed the response button when perceiving the stimulus, which caused the stimulus intensity to decrease (arrows), and when the sensation vanished, participants released the button, which caused the intensity to increase. **(D)** Two out of 20 tactile oddball-detection trials consecutively performed during a scanning session including index (Ind) and little (Litt) finger stimulation. Standard trials contained five 1.5 s pulses with 20 Hz stimulation and in oddball trials, one of the 20 Hz pulses was replaced with a 40 Hz pulse as shown in the second trial illustrated. The participants pressed the response button when perceiving the deviant pulse. **(C,D)** During rest periods between trials, a black cross was shown on the screen. A circle that appeared 3–4.5 s before the start of a tactile trial cued the participants about the upcoming trial. The location of the circle either at the top or bottom of the cross indicated that stimuli will occur at the index or the little finger, respectively. The cue for an upcoming visual trial was a filled circle that substituted the cross. Figure is modified, with permission from Nordmark et al. (2012) published by the MIT Press.

A custom-made equipment could deliver precise tactile stimulations of fingertips of either the left or right hand during MR scanning (Nordmark et al., 2012). It consisted of a rectangular box that that contained tactile stimulators and was anchored to a wooden frame that extended above the participant’s hips in either of two positions, one suitable for a left-hand grasp and one for a right-hand grasp without causing substantial changes in the posture of the arms (Figure 1A). The participants grasped and held the box such that the fingertips of the engaged hand contacted the vertical surface of one side of the box and the thumb contacted the opposing surface (width of the box = 4.9 cm). The foot contralateral to the stimulated hand was strapped in a position that allowed the big toe to operate the push button through which the participant reported their sensations (Figure 1A).

The stimuli were sinusoidal perpendicular movements of a 2-mm wide and 40-mm long ridge contacted by the tip of the either the index or little finger (Figure 1B). The ridge protruded 1 mm above the flat surface of the box and extended in the proximal–distal direction of the fingertip. Thus, the ridge contacted the skin throughout the movement cycles. Bars parallel to the stimulating ridge guided the positioning of the fingers such that they made appropriate contact with the stimulating ridges. The stimulating ridge was set in motion by two coupled moving coils that generated a Lorentz force induced by the large static magnetic field of the MR scanner (Graham et al., 2001; Riener et al., 2005). Current to the coils was provided by a battery-powered power amplifier (Class AB) located in a shielded box in the scanning room and the two matched coils were electrically and mechanically connected so that they rotated in opposite directions to counterbalance the induced voltages from the gradient coils. A low pass pi-filter located at the output of the amplifier prevented interference from high-frequency MR pulses. A lever transferred the torque generated by the rotating coils to the center of the ridge (maximum displacement: ±3 mm; frequency range: 0–100 Hz). To prevent variations in viscoelastic properties between different digits from significantly influencing the relationship between the motor current and ridge position, the coils were loaded by a stiff spring (4 N/mm ridge displacement). The amplitude of the attenuation of ridge movements caused by fingertips loading the stimulators was <10% (for details, including calibration procedures, see Nordmark et al., 2012). A microcomputer located outside the scanning room, connected *via* optical fibers to the power amplifier unit and to the push button in the scanning room, controlled the tactile stimulus parameters and visual display, and administered the experimental protocol.

### Tasks

#### Tactile and Visual Threshold-Tracking Task

In the tactile threshold-tracking task, the ridge oscillated at 20 Hz for 20 s during each trial. The participants continuously reported their awareness of the ridge movement using a variant of the von Bekesy’s threshold-tracking method (Bekesy, 1947; Nordmark et al., 2012; Figure 1C, tactile threshold-tracking trial). The participants indicated that they perceived the ridge moving by pressing a response button, which caused the stimulus amplitude to decrease. When they no longer perceived the stimulation, they released the button, which caused the stimulus amplitude to increase. For each participant and stimulation site, we defined the detection threshold as the midpoint between the median value of the amplitudes recorded at all button presses (upper threshold limen) and one at all button releases (lower threshold limen).

To prevent participants from simply controlling the stimulus amplitude around some arbitrary value by rhythmically pressing the button, we varied the rate of change of the amplitude during the threshold-tracking in a manner unpredictable to the participants. In addition, an adaptive algorithm tuned the rate of amplitude change to normalize the number of button-presses across trials to around four per trial. To that end, first, the change of amplitude between button actions occurred at three different rates (uniform random distribution) that in the first trial corresponded to a doubling/halving of the amplitude during 0.8, 1.1, and 1.6 s, respectively. Second, while keeping the same ratio between the rates, in the subsequent trials conducted with stimulation of the same finger (see further below), the doubling/halving time of the amplitude was adjusted by a coefficient computed as 4 divided by the number of button presses in the previous trial multiplied by the coefficient used in the previous trial (Nordmark et al., 2012). In the first trial with a finger, the starting amplitude of the adaptive algorithm was set to 20 μm peak-to-peak, and in subsequent trials with the same finger it was set to the mean of the amplitudes recorded in the previous trial. On average, the participants performed 4.3 ± 0.5 button presses per tactile threshold-tracking trial (mean ± SD of median values for each participant computed across all four stimulation sites).

Each tactile threshold-tracking trial was followed by a visual threshold-tracking trial, i.e., tactile and visual threshold-tracking trials were interleaved (Figure 1C). During the visual trials, each lasting for 20 s, the participants’ task was to detect a dark circular image displayed in the center of the computer screen against a white background. The visual image, oriented in the plane of the screen, corresponded to a dark circle with uneven color saturation. The diameter was 2° visual angle and its darkness (gray scale) was most saturated in the center of the circle and declined linearly towards its perimeter where it was zero. The color saturation decreased and increased when the pushbutton was pressed and released, respectively. The rate of change of the color saturation and the frequency of button presses was controlled using the same algorithm as for the changes of the amplitude of the ridge movement in the tactile trials. That is, the color saturation decreased and increased when the participants pressed and released the push button, respectively. On average, the participants performed 4.3 ± 0.4 button presses per visual threshold-tracking trial. Importantly, during the visual trials, the participants received the same tactile stimulation as experienced during the previous tactile trial. That is, during each tactile trial, the ridge movement was recorded by the microcomputer system and then replayed to the fingertip in the subsequent visual trial.

Before the first trial and after each tactile and visual trial there was a 14 s period of rest during which a thick solid black cross (1.0° visual angle) was displayed in the center of the otherwise white screen. Before each tactile trial, the participants were cued about the upcoming trial and the finger receiving the stimuli by a black hollow circle appearing above or below the horizontal line of the cross; with the circle above and below the stimulation would be at the index and the little finger, respectively. The cue was shown for 3–4.5 s (uniform random distribution). During tactile trials, the screen showed a thin black cross. When the tactile trial ended, the lines of the cross switched from thin to thick, which indicated the onset of the rest period. A filled black circle (~1.0° visual angle) shown for 3–4.5 s cued the participants about the start of the visual trials.

#### Tactile Oddball-Detection Task

During the tactile oddball-detection task, a standard trial lasted for 10 s. It involved five consecutive pulses consisting of sinusoidal ridge movements at 20 Hz each lasting for 1.5 s and the intervals between the pulses were 0.625 s (Figure 1D; standard trial). The amplitude of the ridge movement was for each tested finger constant at four times the threshold measured in the tactile threshold-tracking task, which was performed before the oddball task. In a pilot experiment on four of the nerve-injured participants, we found that the chosen suprathreshold stimulation was perceived similarly intensive with the affected hand’s index finger as with the little finger of the same hand. The little finger, whose peripheral innervation was unaffected, was constantly stimulated with an amplitude of 4× perception threshold, while the participants controlled the amplitude of a simultaneous 20 Hz stimulation of the index finger with the task of experiencing it as strong as the little finger stimulation. This was done in a replica of the apparatus used in the tactile threshold tracking task. For the four participants, the resulting amplitude ranged between 3.6 and 4.8 times the threshold measured for the index finger, indicating that the stimulus strength was approximately equalized for perceived intensity between the fingers even though the index finger was reinnervated.

Randomized across three out of 10 trials performed with stimulation of each finger (see further below), an oddball stimulation pulse replaced either the second, third, or fourth 20 Hz stimulation pulse in an unpredictable manner. During the oddball pulse the ridge oscillated at 40 Hz for 1.5 s at the same amplitude as during the standard pulses (Figure 1D, oddball trial). The participants were instructed to press the response button once a deviant pulse was detected. A response was considered correct only if a button press occurred within 2.5 s after the onset of a deviant stimulus. The participants received feedback in the earphones on correct and incorrect button presses by a prerecorded voice saying “good” and “oh-oh,” respectively. The rest periods between trials and the cueing of the forthcoming trial and the finger to be engaged had the same structure as for the tactile threshold-tracking trials except that the cueing of the finger to be stimulated was a filled rather than hollow black circle (see Figure 1D).

### General Procedure

Counterbalanced across participants, each participant was engaged in two consecutive MRI scanning sessions, one with stimulation of the left and one with stimulation of right index and little finger. The participant could stand up and move around in the scanner room while the apparatus was rearranged between the sessions. During a scanning session, the participants first performed 24 tactile/visual threshold-tracking trials, 12 with each finger, and then 20 oddball-detection trials, 10 with each finger. The order of stimulation of the two fingers was for each task randomized with the constraint that the same finger could not be involved in more than two consecutive trials. The participant was informed of the type of the upcoming task *via* text on the screen and verbally *via* the headphones. The participants were instructed to look at the computer screen and to keep the fingertips in contact with the tactile stimulator throughout the scanning sessions. Either on the same or the day before the MR-scanning, the participants had learned and practiced the tasks and rehearsed the experimental protocol in a replica of the apparatus.

### MRI Parameters and Data Analysis

Functional MR-images were acquired with a gradient echoplanar imaging sequence (32 transaxial slices, thickness: 4.5 mm, gap: 0 mm, TR: 2,000 ms, TE: 30 ms, flip angle: 80°). The field of view was 25 × 25 cm and contained a 128 × 128-pixel matrix, giving voxels of 1.95 mm × 1.95 mm × 4.5 mm. At the start of each scanning session 10 surplus scans were collected for progressive saturation of the fMRI signal before acquisition of the experimental images. High-resolution T1- weighted structural images were collected with a 3D fast spoiled gradient echo sequence (180 slices with a 1 mm thickness, TR: 8.2 ms, TE: 3.2 ms, flip angle: 12°, field of view: 25 × 25 cm).

The sampled BOLD signals were exported to an off-line Linux-based workstation and converted to NIfTI format and then analyzed with SPM8 (The Wellcome Department of Cognitive Neurology, London, UK^1^). Slice timing correction to the first slice was performed using SPM8’s Fourier phase shift interpolation. The obtained volumes were realigned to the first volume and unwarped to correct for head movements, and then normalized to the standard Montreal Neurological Institute (MNI) EPI template to allow group analysis (2 × 2 × 2 mm^3^ voxel size) after smoothed with an isotropic Gaussian kernel of 6 mm (FWHM). High-pass filtering (128 s period) reduced participant-specific drifts in the BOLD signal and proportional grand mean scaling applied over each scanning session reduced effects of slow global changes in BOLD activity. Batching of analyses, visualization, and extraction of parameter estimates across clusters were performed with software developed in-house (DataZ).

For data obtained in each scanning session, we defined “boxcar” regressors that modeled the various functional states of the participants during the session. Four regressors (20 s) represented the different threshold-tracking trials (tactile trials with stimulation of either index or little finger and the corresponding visual trials) and four regressors (3.0–4.5 s) represented the matching cue periods. In addition, two regressors (10 s) represented the oddball-detection trials with stimulation of either index or little finger and two regressors (3.0–4.5 s) the matching cue periods. To capture residual movement-related artifacts, the six spatial realignment parameters were modeled as covariates. After convolving the boxcar regressors with the standard canonical hemodynamic response function provided by SPM8, a general linear model was fitted to the data obtained for each participant.

In our main analysis, the resulting single-subject images representing the tactile threshold-tracking trials and oddball-detection trials were entered into random effects full factorial mixed-design analysis of variance models (ANOVAs) using SPM8 software, each ANOVA (F-test) allowing two-factor voxel by voxel analysis at a time. A separate whole-brain ANOVA was run on data from each of the four stimulation sites (left and right index and little finger). In these ANOVAs we used task (tactile threshold-tracking task, tactile oddball-detection) as within-subject factor and injury (injured vs. control participants) as between subject factor. To protect against false positives while at the same time retain the power to detect statistically significant effects in these analyses, we subjected the statistical images of the group analysis to a double-threshold approach, in which we combined a voxel-based threshold with a minimum cluster size (Forman et al., 1995). Thresholds were set at an individual voxel threshold of *p* < 0.005, combined with a cluster size threshold of *p* < 0.05. This cluster threshold, corrected for multiple comparisons across the whole brain, was determined based on random field theory (Cao, 1999; Worsley et al., 1999, 2002) and implemented with the stat_threshold function of fmristat available at: http://www.math.mcgill.ca/keith/fmristat. Corresponding analyses were also performed for the cue period.

To spatially relate possible effects of median nerve injury to the somatotopic representations of the stimulated fingers in the contralateral S1, we mapped these representations in a separate voxel-by-voxel ANOVA by directly contrasting the BOLD signals obtained with stimulation of the index and little finger within each hand (F-contrast). In these ANOVAs, one run for each combination of tactile task (tactile threshold-tracking, tactile oddball-detection) and hand (left and right), we also used injury as a fixed effect. Since, we restricted these analyses to the pre- and postcentral gyrus, we decided to use a cluster extent threshold of 20 continuous voxels combined with the voxel threshold of *p* < 0.005.

To explore the signs and sizes of effects detected in the SPM analyses, we extracted for each study participant and experimental condition the β-values across all the voxels of each identified area. The percent BOLD signal change was computed by dividing condition specific β-values with the constant term multiplied by 100. Some of these data were analyzed based on mixed design ANOVAs (*F*-tests) with injury as between-subjects factor (injured vs. control participants) and with within-subject factors as specified in the “Results” section. For *post hoc* analyses we used the Tukey HSD *post hoc* test. Overall, we used an alpha level of 0.05. We report the standardized effect sizes using Cohen’s *d*, which describes the effect size as the difference between the means of the compared groups divided by the pooled standard deviation. For within-group comparisons we used the standard deviation of the differences between pairs of repeated measures.

Based on coordinates provided in the MNI stereotaxic space, the anatomical location of detected clusters and their local maxima were initially assessed by the Automated Anatomical Labeling software (Tzourío-Mazoyer et al., 2002) and cross-referenced to the major sulci and gyri using the stereotactic atlas of Talairach and Tournoux (1988). We then validated this method of localization by superposing the SPMZ maps on study specific mean anatomical T1 image calculated after all participants’ images had been transformed into the MNI stereotactic space. Local maxima located within 10 mm of statistically more significant local maxima were ignored.

The raw data supporting the conclusions of this manuscript will be made available by the authors, without undue reservation, to any qualified researcher. However, in accordance with patient confidentiality, personal data from participants in the study will not be disclosed.

## Results

### Participants’ Performance in the Scanner

**Figure 2:**
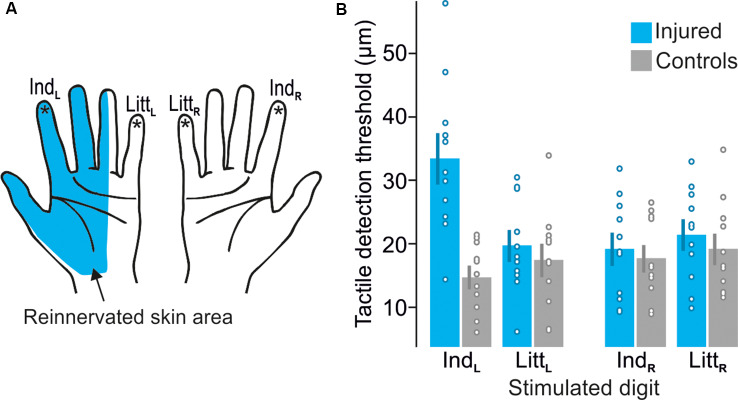
Test sites and detection thresholds recorded during the tactile threshold-tracking task. **(A)** Schematic illustration of the reinnervated skin area after median nerve transection and repair. Stars indicate tested sites: left index (Ind_L_), left little (Litt_L_), right index (Ind_R_) and right little (Litt_R_) fingertips. **(B)** Tactile detection thresholds for median nerve-injured and control participants when stimulating each tested fingertip given as peak-to-peak amplitude of the ridge movement. Heights of columns give mean values computed across participants, error bars indicate ± 1 SEM (*n* = 11) and symbols indicate tactile thresholds for individual participants.

Although our nerve-injured participants had suffered a complete transection of the median nerve directly followed by surgical repair at least 2.5 years before the study, due to usually defective peripheral reinnervation we expected them to exhibit some deficiencies of the tactile sensibility within the reinnervated hand area (Figure 2A). This was confirmed by an elevated tactile threshold for the reinnervated left index finger compared to the control participants as measured in the tactile threshold-tracking task (Figure 2B). For the other tested fingers of either hand innervated by uninjured nerves (left little finger and right index and little finger), the detection thresholds were similar to those of the control participants and similar to thresholds previously reported for 20 Hz oscillatory displacements of fingertip skin (Talbot et al., 1968; Lofvenberg and Johansson, 1984; Nordmark et al., 2012). A mixed design two-way ANOVA run on the tactile threshold values showed a main effect of injury (injured vs. control participants; *F*_(1,20)_ = 4.8; *P* = 0.04), main effect of finger (left and right index and little finger; *F*_(3,60)_ = 5.7; *P* = 0.002) as well as an interaction between injury and finger (*F*_(3,60)_ = 14.4; *P* < 0.0001). *Post hoc* analysis revealed that these effects were driven by a higher threshold at the left index finger of the nerve-injured participants compared to all other test conditions, i.e., combination of participant group and tested finger (*P* ≤ 0.004); there was no significant differences between the other test sites (*P* ≥ 0.49 for all comparisons). Indicated by Cohen’s *d*, the effect size of the threshold increase at the left index finger for nerve injured varied between 1.06 and 1.37 with reference to the threshold of the other tested fingers for the same individuals and it was 1.94 with reference to the control participants’ threshold on the left index finger. We also asked if nerve injury might affect the variation in stimulation intensity relative to the threshold, which was controlled by participants. Assessed by the coefficient of variation calculated for the stimulation amplitudes recorded at all button presses and releases (representing upper and lower threshold limits, respectively) by each participant, we found no effect of injury (*F*_(1,20)_ = 0.03; *P* = 0.87) on this range and no main or interaction effect involving finger (*F*_(3,60)_ ≤ 0.76; *P* ≥ 0.52). Averaged across all participants’ fingers the coefficient of variation was 0.56 ± 0.18 (mean ± SD). Regarding the visual threshold-tracking task, we found no effect on the visual detection threshold of injury (*F*_(1,20)_ = 0.1; *P* = 0.79) and no main or interaction effect involving finger (*F*_(3,60)_ ≤ 0.2; *P* ≥ 0.90). In the tactile oddball-detection task, where the participants received sequences of 20 Hz suprathreshold sinusoidal skin indentations lasting for 1.5 s and their task was to detect a 40 Hz sinusoidal stimulation that occasionally could replace a 20 Hz stimulation (Figure 1D). All participants correctly detected all presented oddballs and there were no false button presses. Taken together, the participants’ performance in the MR scanner during the tactile tasks indicated that they complied with the task instructions and consistently monitored and processed the tactile afferent information.

### Effects of Injury on BOLD Activity

Separate two-factorial whole-brain ANOVAs were used to analyze fMRI data obtained during stimulation of each of the four test sites (left and right index and little finger, Figure 2A). All four ANOVAs revealed brain areas with main effects of injury (injured vs. control participants) and of tactile task (threshold-tracking vs. oddball-detection) but failed to detect any area with interaction between these factors. Four corresponding ANOVAs using functional images from the cue period of the tactile tasks failed to reveal any brain area with effect of injury or task, which suggest that the effects of injury reported below were linked to processing of tactile stimuli rather than just focusing attention on sensing them.

#### Effect of Injury in the Primary Somatosensory Cortex (S1)

**Figure 3:**
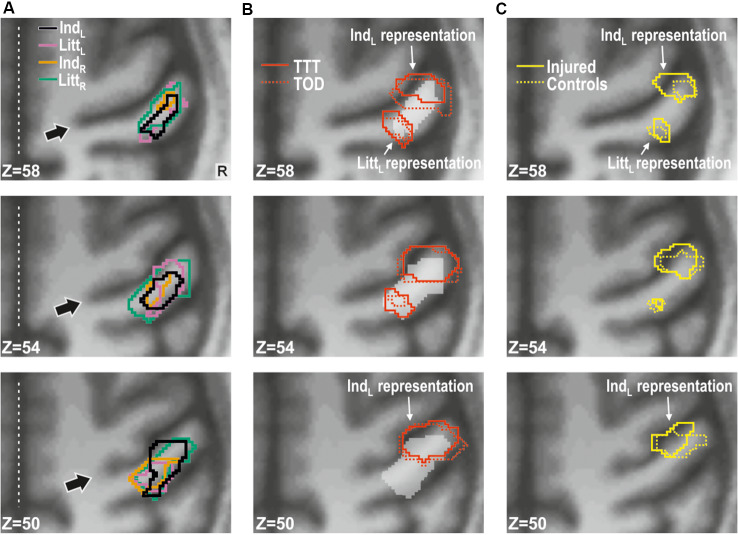
Effect of injury in contralesional S1. **(A)** Main effect of injury obtained with stimulation of each of the fingers shown on transaxial slices of the averaged brain calculated across montreal neurological institute (MNI)-normalized participant-specific T1-weighted images. Lines enclose areas with stronger BOLD activity for the nerve-injured (*n* = 11) compared to the control participants (*n* = 11) and the color of the lines indicate stimulated finger: left index (Ind_L_), left little (Litt_L_), right index (Ind_R_) and right little (Litt_R_) finger. Arrows indicate central sulcus and dotted white lines the interhemispheric fissure. R, right. **(B)** Index finger (Ind_L_) and little finger (Litt_L_) representations outlined based on data from the tactile threshold-tracking task (TTT, red solid lines) and the tactile oddball-detection task (TOD, red dashed lines) performed by all study participants. The white semi-transparent zone represents the envelope of areas with main effects of injury as shown in panel **(A)**. **(C)** Index and little finger representations outlined for injured (yellow solid lines) and control participants (yellow dashed lines) based on the main effect of a finger in ANOVAs separately applied to data from each of the two groups and with finger and task as factors. **(B,C)** Same general format as in panel **(A)**.

For stimulation of each finger, we observed a main effect of injury on the BOLD activity in the contralesional (right) primary sensorimotor cortex and for all fingers this effect was restricted to S1 (Figure 3A). That is, the injury affected brain activity in the contralateral S1 during tactile stimulation within the reinnervated nerve territory (left index finger stimulation) but also in ordinarily innervated cutaneous areas both within the same hand (left little finger stimulation) and within the ipsilateral hand (right index and little finger stimulation). In all cases, the BOLD activity was higher for the nerve-injured group of participants than for the controls. The areas detected during stimulation of the various fingers overlapped highly and involved the anterior bank of the post-central gyrus with some extent toward the crown of the post-central gyrus. Each area showed two local maxima for the BOLD effect in Brodmann area 3 (BA 3; Table 1). There was no main effect of tactile task within S1 regardless of which finger received the tactile stimuli. Further, we could not detect an effect of injury or tactile task in the primary motor cortex (pre-central gyrus) regardless of stimulated finger, and even if we did not apply a cluster size threshold.

**Table 1:** Main effect of injury in contralesional S1.

Stimulated finger	# of voxels	Gyrus	Ze	*X*	*Y*	*Z*
Left index	348	Right Postcentral (BA 3)	3.6	48	−26	54
			3.3	38	−22	44
Left little	289	Right Postcentral (BA 3)	3.9	48	−22	50
			3.1	44	−32	58
Right index	265	Right Postcentral (BA 3)	3.5	40	−28	48
			2.6	50	−22	50
Right little	417	Right Postcentral (BA 3)	3.6	42	−22	44
			3.5	50	−22	50

*The number of contiguous voxels (voxel size = 2 × 2 × 2 mm^3^) is given for identified clusters with the main effect of injury in contralesional S1 in the whole-brain analyses run on data obtained with stimulation of each finger. The brain region, and Brodmann area (BA) refer to coordinates (X, Y, Z; provided in MNI stereotaxic space) of peak Z equivalent (Ze) values located within each cluster. All effects represented higher BOLD activity for the nerve-injured compared with control participants during the execution of the tactile tasks*.

In order to spatially relate the injury effect in the right S1 to its finger representations, we located these by contrasting the BOLD responses obtained during stimulation of the left index and little finger as within-subject effect in an ANOVA applied to each of the tactile task; injury (injured vs. control participants) constituted a between-subject factor. For both tactile tasks, we found two areas with effect of stimulated finger: one area that showed higher BOLD activity during index finger stimulation compared to little finger stimulation and a more posteromedial area that showed higher activity during little finger stimulation than during index finger stimulation. These finger representations derived from the threshold-tracking task and the oddball-detection task overlapped greatly (Figure 3B) and all had their local maxima in BA 3 (Table 2). The Euclidian distance between the index and the little finger representations represented by the local maximum with highest Ze-value exposed by the threshold-tracking and the oddball-detection task was 13.1 mm and 11.0 mm, respectively, which matches previously reported data on finger representations in S1 (van Westen et al., 2004; Martuzzi et al., 2014). Interestingly, we found no voxels that showed an interaction between injury and stimulated finger, which suggests that the regained finger presentations after reinnervation largely resembled the situation before the injury. This was verified in ANOVAs with finger and tactile task as factors separately applied on data from nerve-injured and control participants and with the same threshold criteria as in our main analysis of finger representations as described above (Figure 3C).

**Table 2:** Finger representations in S1 contralateral to stimulated hand.

Finger representation	Task	# of voxels	Gyrus	Ze	*X*	*Y*	*Z*
Left index (Ind_L_ > Litt_L_)	TTT	626	R Postcentral (BA 3)	5.8	48	−14	52
				5.8	46	−16	50
	TOD	607	R Postcentral (BA 3)	6.2	44	−16	56
				6.1	42	−18	52
				4.9	54	−16	60
Left little (Litt_L_ > Ind_L_)	TTT	93	R Postcentral (BA 3)	4.0	42	−24	58
				3.1	50	−28	62
	TOD	47	R Postcentral (BA 3)	4.0	40	−26	58
Right index (Ind_R_ > Litt_R_)	TTT	309	L Postcentral (BA 3)	6.2	−50	−18	52
	TOD	486	L Postcentral (BA 3)	5.2	−50	−16	62
				4.6	−44	−20	52
Right little (Litt_R_ > Ind_R_)	TTT	61	L Postcentral (BA 3)	3.9	−44	−26	62
	TOD	56	L Postcentral (BA 3)	3.5	−40	−30	62

*The number of contiguous voxels (voxel size = 2 × 2 × 2 mm^3^) given for each identified cluster in the primary sensorimotor cortex based on the main effect of the stimulated finger on BOLD activity in scanning sessions with left- and right-hand stimulation. TTT, tactile threshold-tracking task; TOD, tactile oddball-detection task. Hemisphere (L, left; R, right), brain region and Brodmann area (BA) refer to coordinates (X, Y, Z; provided in MNI stereotaxic space) of peak Z equivalent (Ze) values located within each cluster*.

**Figure 4:**
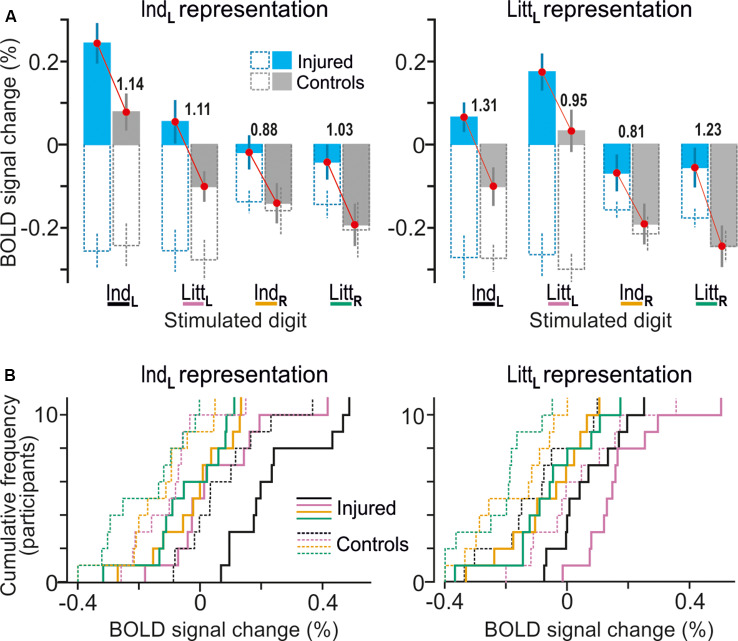
Effects of injury on BOLD responses in fingertip representations in contralesional S1. **(A)** Paired neighboring columns show BOLD signal responses in the identified representations of the index finger (Ind_L_; left panel) and little finger (Litt_L_; right panel) representations when the left index (Ind_L_), left little (Litt_L_), right index (Ind_R_) and right little (Litt_R_) finger was stimulated. Filled columns show BOLD signal responses for the nerve-damaged (bluish) and control participants (gray) based on β-values averaged across all voxels within the respective finger representation and across both tactile tasks (column height indicate mean value across participants and vertical lines represent ± 1 SEM, *N* = 11). To highlight the systematically greater BOLD activity of the nerve-injured irrespective of stimulus conditions, red lines link the corresponding mean values of the injured and the control participants. The adjacent numbers give a standardized effect size for the difference between these mean values provided as Cohen’s *d*. Superimposed hollow dashed-line columns show the corresponding BOLD activity during the visual threshold tracking task for the nerve-injured (bluish) and controls (gray) upon stimulation of each of the four fingers. **(B)** Distribution of BOLD-signal changes across nerve-injured (solid lines) and control participants (dashed lines) during the tactile tasks based on the same data and following the color code for stimulated finger as indicated in panel **(A)**.

For both the left index and little finger representations in right S1, our control participants showed negative BOLD activity when the other tested finger of the same hand received tactile stimuli (Figure 4A, filled gray bars representing Ind_L_ and Litt_L_ stimulation). This is consistent with previous results showing that the topographically organized finger representations in S1 are subject to complex lateral, mainly inhibitory, intra-areal interactions (Ruben et al., 2006; Lipton et al., 2010; Reed et al., 2010; Thakur et al., 2012; Martuzzi et al., 2014). For the controls, the BOLD activity in these representations also showed negative values when the fingers of the right hand received the tactile stimuli (Figure 4A, filled gray bars representing Ind_R_ and Litt_R_ stimulation), which is consistent with previous observations on BOLD responses in S1 during ipsilateral hand stimulations in healthy adults (Hlushchuk and Hari, 2006; Klingner et al., 2015; Tal et al., 2017). A dominant explanation is that signals from S1 contralateral to a stimulated hand are transmitted to the ipsilateral hemisphere *via* callosal connections between higher order somatosensory areas (BA 1, 2 and secondary somatosensory cortex) and feedback connections to area 3b mediate the suppressive effects through local activation of inhibitory neurons (Tommerdahl et al., 2006; Reed et al., 2011; Klingner et al., 2015).

For the nerve-injured participants and for each of the fingers that were stimulated, the group average values of the BOLD activity extracted both within the index and little finger representations were higher compared to the corresponding activity of the controls (Figures 4A,B, see adjacent pairs of filled bluish and gray columns in Figures 4A). As such, this finding harmonizes with the fact that both finger representations in the right S1 essentially overlapped with the area affected by injury according to the whole-brain analysis (Figure 3B, see outlined areas in red with the white semi-transparent zone). Based on the average BOLD activity recorded during the two tactile tasks, the injury had a significant effect on both the identified index and the little finger representation (*F*_(1,20)_ ≥ 10.5; *P* ≤ 0.004 in either case) in addition to the effect of stimulated finger (*F*_(3,60)_ ≥ 32.53; *P* < 0.0001). Neither for the index nor for the little finger representation was there an interaction between stimulated finger and injury (*F*_(3,60)_ ≤ 0.50, *P* ≥ 0.68). Thus, for both identified finger representations in right S1, the increase in BOLD activity of the nerve-injured compared to the control participants was similar regardless of which finger was stimulated (Figure 4A, see lengths of red lines joining corresponding mean values of the injured and control participant). Also, the effect sizes were similar in both finger representations (panel **A**). For the index finger representation, Cohen’s *d* was 1.14 and 1.11 when stimulating the left forefinger and little finger, respectively, and 0.88 and 1.03 for stimulation of the right forefinger and little finger. Corresponding effect sizes for the little finger representation were 1.31, 0.95, 0.81 and 1.23, respectively. We justified the aggregation of data from the two tactile tasks in this analysis by the fact that the whole-brain analysis did not reveal an effect of task in the hand area of S1 no matter which finger received the stimulation and even if the cluster size threshold was reduced to 20 voxels.

Concerning the representations in the left S1 of the right-hand fingers, we found a similar topography as in the right S1, involving distinct representations of the index and the little finger and a high overlap of the representations derived from the two tactile tasks (Table 2). In agreement with the whole-brain analysis, injury had no significant effect on BOLD activity extracted from these finger representations. That is, with our experimental paradigm involving rather strict tactile tasks, standardized stimulation intensities and controls matched to age, gender and handedness, we could not verify previous observations that median nerve-injured persons also show higher BOLD activity in the ipsilesional somatosensory cortex (Chemnitz et al., 2015; Bjorkman and Weibull, 2018).

*Ordinary “task-modality” modulation of S1 after injury*. We asked if the effects of injury observed in the identified finger representations of the contralesional S1 were linked to the use of tactile input as required during the tactile tasks. To that end, we also extracted BOLD signals from these representations when our study participants performed the visual threshold-tracking task. During the visual trials, the participants reported their perception of an image appearing in the center of the computer screen against white background while they received a playback of the tactile stimulus experienced in the previous tactile threshold-tracking trial (Figure 1C). During tactile stimulation of the left fingers, the control participants showed a marked suppression of the BOLD activity for both the index and the little finger representations of right S1 during the visual trials compared to that in the corresponding tactile trials (Figure 4A, see corresponding filled and hollow gray columns representing Ind_L_ and Litt_L_ stimulation). A deactivation also occurred with stimulation of the right fingers but of similar size as that in the corresponding tactile trials (Figure 4A, see corresponding filled and hollow gray columns representing Ind_R_ and Litt_R_ stimulation). Strikingly, despite causing consistently higher BOLD activity during the tactile trials, injury did not appear to influence the suppression of the BOLD signal during the visual trials regardless of finger representation and stimulated finger (Figure 4A, see adjacent pairs of hollow bluish- and gray-line columns). Indeed, mixed-design 3-way ANOVAs run on the BOLD activity recorded during visual trials failed for both representations to indicate effect of injury (*F*_(1,20) ≤_ 0.984, *P* ≥ 0.333) and stimulated finger (index, little finger; *F*_(1,20)≤_ 1.43. *P* ≥ 0.246) but indicated a significant effect of stimulated hand (left, right; *F*_(1,20)_ ≥ 6.47, *P* ≤ 0.019 in either case). As such, for either group of participants the deactivation during the visual task appeared deeper when the contralateral left as compared to the ipsilateral right hand received the tactile stimuli. None of these ANOVAs showed any interaction effect (*P* ≥ 0.30 in all instances). Thus, a seemingly ordinary “task-modality” dependent modulation of the BOLD activity was present in contralesional S1 of the nerve-injured participants.

#### Effect of Injury on Prefrontal Cortex

**Figure 5:**
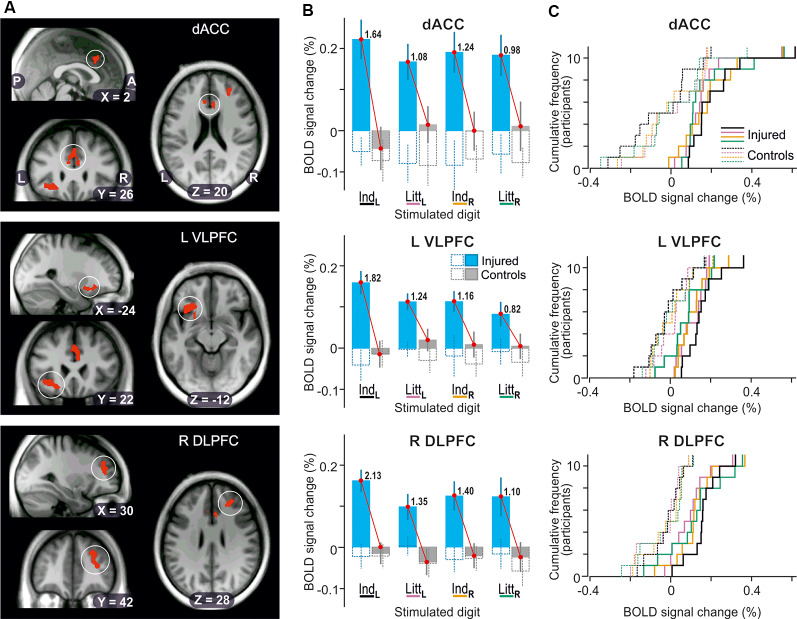
Effect of injury on BOLD responses in the prefrontal cortex. **(A)** Areas with contiguous voxels (voxel size = 2 × 2 × 2 mm^3^) showing* the* main effect of injury detected during stimulation of the left index finger projected on slices of the participant-specific brain image. Top: encircled zones indicate area (218 voxels) with two local maxima in dorsal anterior cingulate cortex (dACC; *x*, *y*, *z* = 6, 22 26, Ze = 3.3; −6, 28, 22, Ze = 3.1; BA 24). Middle: Area (273 voxels) with two local maxima in left ventrolateral prefrontal cortex and insula (L VLPFC; *x*, *y*, *z* = −24, 26, −14, Ze = 3.4; −36, 22, −10, Ze = 3.4, BA 47). Bottom: Area (244 voxels) with two local maxima in right dorsolateral prefrontal cortex (R DLPFC; x, y, *z* = 26, 38, 28, Ze = 3.5; 32, 42, 14, Ze = 3.2, BA 46). P, posterior; A, anterior; L, left; R, right. **(B)** Columns show BOLD-signal responses to stimulation of each of the four fingers tested based on β-values averaged over all voxels of the areas indicated in panel **(A)**. For further format details, see Figure 4A. **(C)** Distribution of BOLD-signal changes across nerve-injured (solid lines) and control participants (dashed lines) during the tactile tasks based on the same data and following the same color code for stimulated finger as indicated in panel **(B)**.

The whole-brain ANOVAs, one run for stimulation of each of the four test sites, also detected main effects of injury in three areas of the prefrontal cortex when the reinnervated left index finger was engaged in the tactile tasks (Figure 5A): the dorsal anterior cingulate cortex (dACC, BA 24); the left ventrolateral prefrontal cortex (VLPFC) on the orbital part of inferior frontal gyrus (BA 47); and the right dorsolateral prefrontal cortex (DLPFC) in the middle frontal gyrus (BA 46). All these areas showed greater BOLD activity in the nerve-injured compared to the control participants with effect sizes ranging between 1.64 and 2.13 (Figure 5B; Ind_L_ stimulation). For each area, stimulation of the other three fingers resulted in similar BOLD signal changes as with left index finger stimulation (Figures 5B,C; Ind_L_, Ind_R_ and Litt_R_ stimulation) but the cluster sizes (81–15 voxels) did not meet the cluster-threshold criterion of the whole-brain analysis. Also, the effect sizes tended to be lower than with left index finger stimulation (Figure 5B). For all four fingertips tested, the BOLD activity of the control subjects was on average close to the baseline (Figures 5B,C, gray filled columns in panel **B)**.

Irrespective of stimulated finger, injury appeared to not influence the BOLD activity recorded during the visual threshold-tracking trials in the detected prefrontal areas (Figure 5B, see adjacent pairs of hollow bluish- and gray-line columns). For none of the areas, a two-way ANOVA indicated an effect of injury (*F*_(1,20)_ ≤ 0.31, *P* ≥ 0.58), of finger (*F*_(3,60)_ ≤ 0.27, *P* ≥ 0.85), or interaction between injury and finger (*F*_(3,60)_ ≤ 0.60, *P* ≥ 0.62). Only for dACC was the intercept significantly different from zero (*F*_(1,20)_ = 7.57; *P* = 0.012), which indicated that the BOLD activity overall tended to be below baseline values during the visual trials.

### Effects Related to Tactile Task

Each whole-brain ANOVA with injury and tactile task as factors revealed effects of task in a network of brain areas. These areas corresponded to those we previously attributed to processes linked to the control of the action of the foot contralateral to the hand involved in the task when healthy adults perform the tactile threshold-tracking task (Nordmark et al., 2012). Briefly, irrespective of stimulated finger we found a greater BOLD activity during the tactile threshold-tracking task than in the oddball-detection task in the foot area of the primary sensorimotor cortex contralateral to the foot operating the pushbutton. Likewise, greater BOLD activity was observed in the contralateral premotor cortex and putamen, and in the ipsilateral anterior foot area of the cerebellum. Furthermore, increased BOLD activity irrespective of stimulated finger were present bilaterally in the supplementary motor area and in the right ventral premotor area. We attribute all these effects to the fact that the frequency of button-presses was around an order of magnitude higher during the tactile threshold-tracking task (mean = 4.3/20 s) than during the oddball-detection tasks (0.3/10 s).

### Additional Observations on BOLD Activity

In addition to addressing our principal topic, i.e., effect of median nerve injury on BOLD activity, in a separate conjunction analysis we also explored brain regions with increased BOLD activity relative to rest during the tactile tasks irrespective of stimulated finger, task and injury. The results were virtually indistinguishable from those of a corresponding analysis we previously reported on for the tactile threshold tacking task performed by healthy adults (Nordmark et al., 2012). Briefly, bilateral activation was present in the perisylvian cortex including the secondary somatosensory cortex, the superior temporal cortex, inferior parietal lobule, supramarginal gyrus and insula. Bilateral activation was also present in the mesial frontal cortex (including pre-SMA and anterior cingulate cortex) and in the putamen and palladium of basal ganglia. Particularly extensively for the right hemisphere, prefrontal activity engaged the anterior precentral gyrus and extended laterally to the posterior part of the VLPFC and insula. Activity was also observed in the right DLPFC. Activated regions in the prefrontal cortex and basal ganglia have often been implicated in cognitive functions such as attention, decision making and response selection (Bunge, 2004; Seeley et al., 2007; Heekeren et al., 2008; Romo et al., 2012) which all were components of both the tactile threshold-tracking and the oddball-detection tasks.

## Discussion

Our central finding is that the hand representation of the right primary somatosensory cortex (S1) of the left median nerve-injured participants showed an elevated BOLD activity compared to that of the controls not only when the left reinnervated index finger was engaged in the tactile tasks, but also when fingers innervated by uninjured nerves of either hand were used, i.e., the left little finger and the right index and little finger. Likewise, the contralesional S1 of the injured participants basically lacked the negative BOLD responses within the identified finger representations as observed for the controls during stimulation of all fingers except for the represented finger. Below, we will argue that the overall increased BOLD activity in the contralesional S1 represented a general disinhibition of its hand area consistent with an augmented functional reorganizational plasticity being constantly present in chronic nerve injury. We also found elevated BOLD-activity for the nerve-injured participants in prefrontal cortical areas during execution of the tactile tasks. We interpret this as if top-down processes that support decision-making and response selection were computationally more demanding for the injured participants due to their compromised tactile sensibility compared to the control participants.

### Primary Somatosensory Cortex

Although exactly how cortical BOLD responses are translated into neural activity is still unknown, essentially based on animal studies there is a consensus that regional stimulation- or task-induced increases and decreases of BOLD activity broadly represent corresponding changes in neural activity (Logothetis, 2008; Hillman, 2014) and that increased activity in inhibitory cortical interneurons generally reduces rather than increases BOLD activity and can render BOLD signals negative (Shmuel et al., 2006; Devor et al., 2007; Logothetis, 2008; Lauritzen et al., 2012). Since cortical activity, in a broad sense, reflects the interaction between synaptic excitation and cortex inhibition (Isaacson and Scanziani, 2011), a change in the balance of excitation-inhibition towards excitation would have caused the increased activity in the contralesional S1 during the tactile tasks. Such a change can hardly be explained by an increased incoming excitation based on intensified peripheral afferent input. That is, histological studies in monkeys have shown that the number of functioning first-order tactile neurons typically is reduced compared to healthy conditions (Liss et al., 1996) and recordings from human median and ulnar nerves have shown that reinnervated neurons do not exhibit increased sensitivity and firing rates (Mackel et al., 1983; Mackel, 1985). Nor can functional plastic changes of the somatotopic organization within tactile ascending pathways easily account for an increased incoming excitation of the contralesional S1 since the increase in activity was not restricted to the median nerve projection zone. That is, findings in monkeys subjected to experimental median nerve transection and reinnervation indicate that such plasticity essentially would be confined to median nerve projection areas because in S1 it was confined to its median nerve representation (Wall et al., 1986; Merzenich and Jenkins, 1993). Indeed, any functional somatotopic reorganization of contralesional S1 would have been limited also in our study material, because the locations and extents of the identified finger representations of the nerve-injured and control participants were quite comparable.

If increased incoming excitation cannot readily explain the greater stimulation-induced activity in contralesional S1, then the question is whether intra-cortical mechanisms can. Known intra-cortical mechanisms implicated in regulation and stabilization of cortical excitability include synaptic scaling (or homeostatic scaling; Turrigiano, 2012; Keck et al., 2017) and intra-areal (local) inhibitory processes (Isaacson and Scanziani, 2011). Since synaptic scaling has a normalizing/stabilizing function on the excitatory cortical activity it should have counteracted rather than promoted an increase of activity in contralesional S1. Instead, the greater activity in response to incoming excitation resulted most likely from reduction of stimulus-induced intra-cortical inhibition effected by reduced activity in inhibitory (GABAergic) interneurons recruited *via* feed-forward and/or feedback-excitatory projections (Isaacson and Scanziani, 2011). Indeed, intracortical studies in monkeys have shown that excitatory neurons of S1 are subject to complex lateral inhibitory interactions supported by intra-areal inhibitory neurons (Lipton et al., 2010; Reed et al., 2010, 2011; Thakur et al., 2012). This notion that the nerve injury lead to a disinhibition of the contralesional S1 in our study participants is supported by the finding that the negative BOLD signals, signifying inhibition (Shmuel et al., 2006; Devor et al., 2007; Logothetis, 2008; Lauritzen et al., 2012), observed in S1’s finger representations of the controls were largely reduced or absent in the injured.

Animal studies indicate that reducing the levels of GABAergic inhibition onto excitatory cortical neurons plays a decisive role in plasticity not only during critical periods of development (Hensch, 2005; Takesian et al., 2018) but also for inducing (and maintaining) excitatory cell reorganizations in deprived projection zones of a lesioned peripheral nerve in adulthood (Arckens et al., 2000; Garraghty et al., 1991, 2006; Levy et al., 2002; Marik et al., 2010; Chen et al., 2011; Sammons and Keck, 2015). A reduction of GABAergic inhibition occurs immediately after a peripheral nerve lesion and is considered permissive for unmasking and strengthening of pre-existing synaptic connectivity and possible axonal sprouting, which drives the reorganization in search for new stable circuity states given the available input conditions. Conversely, and importantly, GABA levels gradually increase towards baseline levels when the functional reorganization (newly formed circuit connectivity) stabilizes (Sammons and Keck, 2015). In view of this, we interpret that the elevated BOLD activity in contralesional S1 of the nerve-damaged participants was caused by a reduced stimulation-induced GABAergic inhibition reflecting that this stabilization was not yet completed. Thus, we put forward that a post-lesional reorganizational plasticity was still ongoing in the contralesional S1 of our nerve-injured study participants even though the injury occurred several years earlier (mean = 8.1 years) and the attainable recovery of the hand should have been completed. Our position in this regard finds support in recent results from studied in monkey that indicate changes in AMPA- as well as GABA-receptor expressions in the brain stem and S1 that consistent with an ongoing reorganization plasticity occurring 5 years after median and ulnar nerve transection (Mowery et al., 2015).

We speculate that such ongoing reorganizational plasticity relates to limitations in the Hebbian-based learning rules considered to account for the formation of cortical topographic maps and cell assemblies representing natural stimuli in primate S1 (Buonomano and Merzenich, 1998). Due to the spatiotemporal aberrant (noisy) activity in ensembles of first-order tactile neurons after reinnervation, these rules may partially fail to achieve a complete stabilization of S1 since they are based on the detection of spatiotemporally correlated inputs with precise time criteria and from a limited cutaneous area (Pons et al., 1991; Song and Abbott, 2001; Feldman, 2012). Thus, substantially dispersed peripheral conduction velocities as well as spatially misaligned reinnervation of axons could reduce the scope of correlation-based functional reorganizations within S1. Also reduced motor activity for a long time of the affected hand may have contributed to aberrant spatiotemporally correlated inputs to S1 as it communicates tightly with motor cortex during planning and control of actions (Jones, 1986; Dum and Strick, 1996). In fact, evidence exists that non-structured (noisy) inputs can not only induce but also sustain critical-period-like plasticity in adult rat primary sensory cortex (Chang and Merzenich, 2003; Zhou et al., 2011).

That the BOLD activity during the visual task was suppressed to the same levels in the injured and the control participants (Figure 4A) suggests that this task-modality dependent suppression involved inhibitory mechanisms that differed from those that we propose operating on the contralesional S1 of the nerve-injured participants. Indeed, there is evidence that specific subtypes of inhibitory neurons in primate sensory cortices can perform context-dependent modulation of excitatory activity while others can regulate activity-dependent plasticity of excitatory circuits (Hattori et al., 2017). In a previous study on healthy adults that involved tactile and visual threshold-tracking tasks similar to those performed in this study, we found that the corresponding task modality-dependent effect not only engages S1 but bilateral cortical areas extending from the hand area of the postcentral gyrus laterally into the perisylvian region and further anterior into the anterior insula of the frontal lobe (see Figure 5B in Nordmark et al., 2012). Hence, the effect was likely instantiated by inter-areal “top-down” attentional-related global modulation of somatosensory processing pathways driven by higher-level processing distributed within frontal and parietal cortex, cerebellum and thalamus (Gilbert and Sigman, 2007; Dosenbach et al., 2008).

### Prefrontal Cortex

The three prefrontal areas showing higher activity for the nerve-injured participants during the tactile tasks (Figure 5A) were all located in the close vicinity of prefrontal areas that we previously found to be commonly activated during the tactile threshold tracking task when performed by healthy adults (Nordmark et al., 2012). According to previous human fMRI studies, the detected prefrontal areas are probably components of two separate networks supporting different types of top-down control (Dosenbach et al., 2008): the dACC and VLPFC together with anterior insula and thalamus have been implicated in control of goal directed behavior through the stable maintenance of task sets, while DLPFC together with inferior parietal lobule, intraparietal sulcus, precuneus and middle cingulate cortex in error-related activity and initiation and adaptations of control on a trial-by-trial basis. The greater BOLD activity in the nerve-injured participants suggests that such control processes, central to decision-making and response selection, were more demanding for the injured participants due to their compromised tactile sensibility than for the controls. Furthermore, it cannot be ruled out that the increased activity in these prefrontal brain areas may have reflected execution of top-down influences somehow supporting the proposed disinhibition of the contralesional S1.

The tendency to elevated BOLD activity in the tactile tasks irrespective of stimulated finger might reflect that everyday dexterous actions practically always engage multiple digits and often both hands (Kilbreath and Heard, 2005; Johansson and Flanagan, 2009; Lederman and Klatzky, 2009; Ingram and Wolpert, 2011). That is, provided that decoding and higher-level processing of tactile information are routinely based on spatiotemporally organized inputs from digits of both hands, compromised tactile inputs from one hand’s median nerve territory could affect processing of tactile inputs irrespective of stimulated digit. Furthermore, considering the involvement of orbitofrontal cortex in implementation of stimulus-reinforcement associations in human behavior (Kringelbach and Rolls, 2004), the effect in especially the left VLPFC might relate to processes involved in coping with pain, numbness, stiffness etc. associated with use of the a reinnervated hand in various manual tasks (Irwin et al., 1997; Chemnitz et al., 2013).

### Limitations and General Issues

#### Limitations of the Present Study

Due to the small sample size, our study might have been underpowered to detect relevant effects and might have overestimated detected effect sizes (Button et al., 2013). However, the approach of focusing our analyses to the identified finger representations in S1 should have helped in these respects. Although our way of mapping the fingertip representations in S1 appeared to make sense concerning previous accounts, fMRI at ultra-high field (≥ 7 T) would have offered far greater spatial detail (Martuzzi et al., 2014) and additional information about content-based processing if also combined with recent advances in multivariate pattern analysis (Haynes, 2015). However, there are still limitations on how perfusion-related (BOLD) signals can be interpreted in terms of changes in neural activity (Logothetis, 2008; Hillman, 2014).

Although we did not measure finger contact forces in the MRI scanner during the tactile trials, we do not believe that the detected effects of injury on the BOLD signals were caused by differences between the injured and control participants in their fingers’ contact behavior. First, contact force has little effect on tactile sensitivity at 20 Hz vibrotactile stimulation (i.e., the frequency of the current study), especially when delivered *via* a contactor protruding through a hole in an otherwise flat contact surface (as in our study; Harada and Griffin, 1991; Gu and Griffin, 2012). Such stimuli are primarily encoded by first-order tactile neurons innervating Meissner corpuscles (FA-1 neurons; Johansson et al., 1982; Lofvenberg and Johansson, 1984). Background contact force has little effect on the responsiveness of these neurons since these are primarily excited by local changes in skin strain within their small receptive fields (Phillips et al., 1992), which in our study was caused by the edges of the vibrating stimulation ridge. Second, we did not see an effect of injury in the motor cortex, which one might expect if the injured participants applied a different, computationally more demanding, contact force strategy to sense the tactile stimuli.

#### Strengths of the Present Study

An important strength of the present study was the strict experimental setup. First, the intensity of our tactile stimuli was perceptually equalized across the various test sites, which aimed to standardize the effective afferent input especially to compensate for a reduced sensitivity of the reinnervated index finger. Second, we adopted well-defined tasks that properly attempted to control for and standardize top-down effects related to task/attentional set across participants. We considered this important because the activity of the primary somatosensory cortex can be modulated by the relevance of stimuli to behavior, but also fingertip representations can be shaped by task/attentional set (Braun et al., 2002; Lipton et al., 2010; Kida et al., 2018). A dramatic impact of task requirements on the BOLD-activity in the contralateral S1 was evident in this study when comparing activity evoked during the visual threshold-tracking task and the tactile tasks.

We do not interpret our failure to detect differential effects of our tactile tasks on the BOLD signals in the somatosensory cortical areas as if the neural processing was indifferent to the task, we rather believe that existing task effects did not cause perfusion contrasts detectable by our technique. Yet, it may seem strange that we did not register stronger BOLD responses in S1 in the oddball-detection task in which the stimulus intensity was higher than in the tactile threshold-tracking task. However, a positive relationship between the stimulus intensity and the intensity of the BOLD response has been observed within a given task set or with relaxed subjects (Arthurs et al., 2000; Nelson et al., 2004; Siedentopf et al., 2008), while in our case, the tactile threshold-tracking and oddball-detection tasks represented different task sets. We suggest that this factor, in combination with the canonical neural calculation principle in sensory cortices called divisive normalization, can explain the similarity in BOLD activity in S1 in our two tactile tasks: divisive normalization generates normative coding efficiency *via* processes such as gain control, feature invariance, and redundancy reduction (Carandini and Heeger, 2011; Isaacson and Scanziani, 2011; Brouwer et al., 2015). Incidentally, even microstimulation of individual first-order tactile afferent neurons innervating the human fingers may give rise to robust fMRI responses in the somatosensory cortex (Trulsson et al., 2001).

#### Clinical Relevance

That our results suggest that S1 can be quite open to functional reorganizational plasticity even very late after peripheral nerve injury and reinnervation might help to motivate future scientific efforts to further develop and improve regimen for hand function training during chronic recovery from nerve injury. Possibly even more fruitful would be to longitudinally follow the dynamics of functional brain plasticity from early after injury and correlate it with behavioral, treatment and rehabilitation variables, to find therapeutic regimens that optimize the utilization of this dynamic. In interaction with basic research on underlying neuronal pathophysiological mechanisms, in this context, there are potentially many directions for further development and refinement of monitoring the dynamics of brain plasticity in humans. For example, combinations of high-resolution fMRI techniques and integrated findings from other modalities, such as magnetoencephalography (Mogilner et al., 1993) and GABA-edited magnetic resonance spectroscopy (Puts and Edden, 2012) in longitudinal studies, could contribute in this regard.

## Data Availability Statement

The datasets generated for this study are available on request to the corresponding author.

## Ethics Statement

The studies involving human participants were reviewed and approved by Regionala etikprövningsnämnden i Umeå. The patients/participants provided their written informed consent to participate in this study.

## Author Contributions

PN and RJ designed the study, interpreted and validated the results, prepared the manuscript and contributed to the reviewing of the final version. PN recruited the patients, performed the data sampling and the main analysis.

## Conflict of Interest

The authors declare that the research was conducted in the absence of any commercial or financial relationships that could be construed as a potential conflict of interest.

## Footnotes

^1^http://www.fil.ion.ucl.ac.uk/spm

## References

[B1] Allodi I. Udina E. Navarro X. (2012). Specificity of peripheral nerve regeneration: interactions at the axon level. Prog. Neurobiol. 98, 16–37. 10.1016/j.pneurobio.2012.05.005 22609046

[B2] Arckens L. Schweigart G. Qu Y. Wouters G. Pow D. V. Vandesande F.. (2000). Cooperative changes in GABA, glutamate and activity levels: the missing link in cortical plasticity. Eur. J. Neurosci. 12, 4222–4232. 10.1046/j.0953-816x.2000.01328.x 11122334

[B3] Arthurs O. J. Williams E. J. Carpenter T. A. Pickard J. D. Boniface S. J. (2000). Linear coupling between functional magnetic resonance imaging and evoked potential amplitude in human somatosensory cortex. Neuroscience 101, 803–806. 10.1016/s0306-4522(00)00511-x 11113329

[B4] Bekesy G. V. (1947). The recruitment phenomenon and difference limen in hearing and vibration sense. Laryngoscope 57, 765–777. 10.1288/00005537-194712000-00002 18918245

[B5] Bjorkman A. Weibull A. (2018). Loss of inhibition in ipsilateral somatosensory areas following altered afferent nerve signaling from the hand. Neurosci. Res. 135, 32–36. 10.1016/j.neures.2017.12.004 29258852

[B6] Braun C. Haug M. Wiech K. Birbaumer N. Elbert T. Roberts L. E. (2002). Functional organization of primary somatosensory cortex depends on the focus of attention. NeuroImage 17, 1451–1458. 10.1006/nimg.2002.1277 12414284

[B7] Brouwer G. J. Arnedo V. Offen S. Heeger D. J. Grant A. C. (2015). Normalization in human somatosensory cortex. J. Neurophysiol. 114, 2588–2599. 10.1152/jn.00939.2014 26311189 PMC4637367

[B8] Buchthal F. Kühl V. (1979). Nerve conduction, tactile sensibility, and the electromyogram after suture or compression of peripheral nerve: a longitudinal study in man. J. Neurol. Neurosurg. Psychiatry 42, 436–451. 10.1136/jnnp.42.5.436 448383 PMC490231

[B9] Bunge S. A. (2004). How we use rules to select actions: a review of evidence from cognitive neuroscience. Cogn. Affect. Behav. Neurosci. 4, 564–579. 10.3758/cabn.4.4.564 15849898

[B10] Buonomano D. V. Merzenich M. M. (1998). Cortical plasticity: from synapses to maps. Annu. Rev. Neurosci. 21, 149–186. 10.1146/annurev.neuro.21.1.149 9530495

[B11] Burnett M. G. Zager E. L. (2004). Pathophysiology of peripheral nerve injury: a brief review. Neurosurg. Focus 16:E1. 10.3171/foc.2004.16.5.2 15174821

[B12] Button K. S. Ioannidis J. P. Mokrysz C. Nosek B. A. Flint J. Robinson E. S.. (2013). Power failure: why small sample size undermines the reliability of neuroscience. Nat. Rev. Neurosci. 14, 365–376. 10.1038/nrn3475 23571845

[B13] Cao J. (1999). The size of the connected components of excursion sets of χ^2^, t and F fields. Adv. Appl. Probab. 31, 579–595. 10.1239/aap/1029955192

[B14] Carandini M. Heeger D. J. (2011). Normalization as a canonical neural computation. Nat. Rev. Neurosci. 13, 51–62. 10.1038/nrn3136 22108672 PMC3273486

[B15] Chang E. F. Merzenich M. M. (2003). Environmental noise retards auditory cortical development. Science 300, 498–502. 10.1126/science.1082163 12702879

[B16] Chemnitz A. Björkman A. Dahlin L. B. Rosén B. (2013). Functional outcome thirty years after median and ulnar nerve repair in childhood and adolescence. J. Bone Joint Surg. Am. 95, 329–337. 10.2106/jbjs.l.00074 23426767

[B17] Chemnitz A. Weibull A. Rosén B. Andersson G. Dahlin L. B. Björkman A. (2015). Normalized activation in the somatosensory cortex 30 years following nerve repair in children: an fMRI study. Eur. J. Neurosci. 42, 2022–2027. 10.1111/ejn.12917 25865600

[B19] Chen R. Cohen L. G. Hallett M. (2002). Nervous system reorganization following injury. Neuroscience 111, 761–773. 10.1016/s0306-4522(02)00025-8 12031403

[B18] Chen J. L. Lin W. C. Cha J. W. So P. T. Kubota Y. Nedivi E. (2011). Structural basis for the role of inhibition in facilitating adult brain plasticity. Nat. Neurosci. 14, 587–594. 10.1038/nn.2799 21478885 PMC3083474

[B20] de Ruiter G. C. Spinner R. J. Verhaagen J. Malessy M. J. (2014). Misdirection and guidance of regenerating axons after experimental nerve injury and repair. J. Neurosurg. 120, 493–501. 10.3171/2013.8.jns122300 24116727

[B21] Devor A. Tian P. Nishimura N. Teng I. C. Hillman E. M. Narayanan S. N.. (2007). Suppressed neuronal activity and concurrent arteriolar vasoconstriction may explain negative blood oxygenation level-dependent signal. J. Neurosci. 27, 4452–4459. 10.1523/JNEUROSCI.0134-07.2007 17442830 PMC2680207

[B22] Dosenbach N. U. Fair D. A. Cohen A. L. Schlaggar B. L. Petersen S. E. (2008). A dual-networks architecture of top-down control. Trends Cogn. Sci. 12, 99–105. 10.1016/j.tics.2008.01.001 18262825 PMC3632449

[B23] Dum R. P. Strick P. L. (1996). “The corticospinal system: a structural framework for the central control of movement,” in Handbook of Physiology, Exercise: Regulation and Integration of Multiple Systems, eds Rowell L. B. Sheperd J. T. (New York, NY: Oxford University Press), 217–254.

[B24] Feldman D. E. (2012). The spike-timing dependence of plasticity. Neuron 75, 556–571. 10.1016/j.neuron.2012.08.001 22920249 PMC3431193

[B25] Florence S. L. Garraghty P. E. Wall J. T. Kaas J. H. (1994). Sensory afferent projections and area 3b somatotopy following median nerve cut and repair in macaque monkeys. Cereb. Cortex 4, 391–407. 10.1093/cercor/4.4.391 7950311

[B26] Forman S. D. Cohen J. D. Fitzgerald M. Eddy W. F. Mintun M. A. Noll D. C. (1995). Improved assessment of significant activation in functional magnetic resonance imaging (fMRI): use of a cluster-size threshold. Magn. Reson. Med. 33, 636–647. 10.1002/mrm.1910330508 7596267

[B27] Fornander L. Nyman T. Hansson T. Ragnehed M. Brismar T. (2010). Age- and time-dependent effects on functional outcome and cortical activation pattern in patients with median nerve injury: a functional magnetic resonance imaging study. J. Neurosurg. 113, 122–128. 10.3171/2009.10.jns09698 19911892

[B28] Garraghty P. E. Arnold L. L. Wellman C. L. Mowery T. M. (2006). Receptor autoradiographic correlates of deafferentation-induced reorganization in adult primate somatosensory cortex. J. Comp. Neurol. 497, 636–645. 10.1002/cne.21018 16739196 PMC4139035

[B29] Garraghty P. E. LaChica E. A. Kaas J. H. (1991). Injury-induced reorganization of somatosensory cortex is accompanied by reductions in GABA staining. Somatosens. Mot. Res. 8, 347–354. 10.3109/08990229109144757 1667058

[B30] Gilbert C. D. Sigman M. (2007). Brain states: top-down influences in sensory processing. Neuron 54, 677–696. 10.1016/j.neuron.2007.05.019 17553419

[B31] Gomez-Ramirez M. Hysaj K. Niebur E. (2016). Neural mechanisms of selective attention in the somatosensory system. J. Neurophysiol. 116, 1218–1231. 10.1152/jn.00637.2015 27334956 PMC5018055

[B7100] Graham S. J. Staines W. R. Nelson A. Plewes D. B. McIlroy W. E. (2001). New devices to deliver somatosensory stimuli during functional MRI. Magn. Reson. Med. 46, 436–442. 10.1002/mrm.1211 11550233

[B32] Grinsell D. Keating C. P. (2014). Peripheral nerve reconstruction after injury: a review of clinical and experimental therapies. Biomed. Res. Int. 2014:698256. 10.1155/2014/698256 25276813 PMC4167952

[B33] Gu C. Griffin M. J. (2012). Vibrotactile perception thresholds at the sole of the foot: effects of contact force and probe indentation. Med. Eng. Phys. 34, 447–452. 10.1016/j.medengphy.2011.08.002 21917498

[B34] Hallin R. G. Wiesenfeld Z. Lindblom U. (1981). Neurophysiological studies on patients with sutured median nerves: faulty sensory localization after nerve regeneration and its physiological correlates. Exp. Neurol. 73, 90–106. 10.1016/0014-4886(81)90047-9 7250291

[B35] Hansson T. Brismar T. (2003). Loss of sensory discrimination after median nerve injury and activation in the primary somatosensory cortex on functional magnetic resonance imaging. J. Neurosurg. 99, 100–105. 10.3171/jns.2003.99.1.0100 12854750

[B36] Harada N. Griffin M. J. (1991). Factors influencing vibration sense thresholds used to assess occupational exposures to hand transmitted vibration. Br. J. Ind. Med. 48, 185–192. 10.1136/oem.48.3.185 2015210 PMC1035347

[B37] Hattori R. Kuchibhotla K. V. Froemke R. C. Komiyama T. (2017). Functions and dysfunctions of neocortical inhibitory neuron subtypes. Nat. Neurosci. 20, 1199–1208. 10.1038/nn.4619 28849791 PMC7082034

[B38] Haynes J. D. (2015). A primer on pattern-based approaches to fMRI: principles, pitfalls, and perspectives. Neuron 87, 257–270. 10.1016/j.neuron.2015.05.025 26182413

[B39] Heekeren H. R. Marrett S. Ungerleider L. G. (2008). The neural systems that mediate human perceptual decision making. Nat. Rev. Neurosci. 9, 467–479. 10.1038/nrn2374 18464792

[B40] Hensch T. K. (2005). Critical period plasticity in local cortical circuits. Nat. Rev. Neurosci. 6, 877–888. 10.1038/nrn1787 16261181

[B41] Hillman E. M. (2014). Coupling mechanism and significance of the BOLD signal: a status report. Annu. Rev. Neurosci. 37, 161–181. 10.1146/annurev-neuro-071013-014111 25032494 PMC4147398

[B42] Hlushchuk Y. Hari R. (2006). Transient suppression of ipsilateral primary somatosensory cortex during tactile finger stimulation. J. Neurosci. 26, 5819–5824. 10.1523/JNEUROSCI.5536-05.2006 16723540 PMC6675271

[B43] Hsiao S. S. O’Shaughnessy D. M. Johnson K. O. (1993). Effects of selective attention on spatial form processing in monkey primary and secondary somatosensory cortex. J. Neurophysiol. 70, 444–447. 10.1152/jn.1993.70.1.444 8360721

[B44] Ingram J. N. Wolpert D. M. (2011). Naturalistic approaches to sensorimotor control. Prog. Brain Res. 191, 3–29. 10.1016/b978-0-444-53752-2.00016-3 21741541

[B45] Irwin M. S. Gilbert S. E. Terenghi G. Smith R. W. Green C. J. (1997). Cold intolerance following peripheral nerve injury. Natural history and factors predicting severity of symptoms. J. Hand Surg. Br. 22, 308–316. 10.1016/s0266-7681(97)80392-0 9222907

[B46] Isaacson J. S. Scanziani M. (2011). How inhibition shapes cortical activity. Neuron 72, 231–243. 10.1016/j.neuron.2011.09.027 22017986 PMC3236361

[B47] Jaquet J. B. Luijsterburg A. J. Kalmijn S. Kuypers P. D. Hofman A. Hovius S. E. (2001). Median, ulnar, and combined median-ulnar nerve injuries: functional outcome and return to productivity. J. Trauma 51, 687–692. 10.1097/00005373-200110000-00011 11586160

[B48] Johansen-Berg H. Christensen V. Woolrich M. Matthews P. M. (2000). Attention to touch modulates activity in both primary and secondary somatosensory areas. Neuroreport 11, 1237–1241. 10.1016/s1053-8119(00)90952-2 10817599

[B49] Johansson R. S. Flanagan J. R. (2009). Coding and use of tactile signals from the fingertips in object manipulation tasks. Nat. Rev. Neurosci. 10, 345–359. 10.1038/nrn2621 19352402

[B50] Johansson R. S. Landström U. Lundström R. (1982). Responses of mechanoreceptive afferent units in the glabrous skin of the human hand to sinusoidal skin displacements. Brain Res. 244, 17–25. 10.1016/0006-8993(82)90899-x 6288178

[B51] Jones E. G. (1986). “Connectivity of the primate sensory-motor cortex,” in Cerebral Cortex, Vol 5, Sensory-Motor Areas and Aspects of Cortical Connectivity, eds Jones E. G. Peters A. (New York, NY: Plenum), 113–183.

[B52] Kaas J. H. (1991). Plasticity of sensory and motor maps in adult mammals. Annu. Rev. Neurosci. 14, 137–167. 10.1146/annurev.ne.14.030191.001033 2031570

[B53] Keck T. Toyoizumi T. Chen L. Doiron B. Feldman D. E. Fox K.. (2017). Integrating Hebbian and homeostatic plasticity: the current state of the field and future research directions. Philos. Trans. R. Soc. Lond. B Biol. Sci. 372:20160158. 10.1098/rstb.2016.0158 28093552 PMC5247590

[B54] Kida T. Tanaka E. Kakigi R. (2018). Adaptive flexibility of the within-hand attentional gradient in touch: an MEG study. NeuroImage 179, 373–384. 10.1016/j.neuroimage.2018.06.063 29936309

[B55] Kilbreath S. L. Heard R. C. (2005). Frequency of hand use in healthy older persons. Aust. J. Physiother. 51, 119–122. 10.1016/s0004-9514(05)70040-4 15924514

[B56] Klingner C. M. Brodoehl S. Witte O. W. (2015). The importance of the negative blood-oxygenation-level-dependent (BOLD) response in the somatosensory cortex. Rev. Neurosci. 26, 647–653. 10.1515/revneuro-2015-0002 26057216

[B57] Krarup C. Rosen B. Boeckstyns M. Ibsen Sørensen A. Lundborg G. Moldovan M.. (2017). Sensation, mechanoreceptor, and nerve fiber function after nerve regeneration. Ann. Neurol. 82, 940–950. 10.1002/ana.25102 29156496

[B58] Kringelbach M. L. Rolls E. T. (2004). The functional neuroanatomy of the human orbitofrontal cortex: evidence from neuroimaging and neuropsychology. Prog. Neurobiol. 72, 341–372. 10.1016/j.pneurobio.2004.03.006 15157726

[B59] LaMotte R. H. Mountcastle V. B. (1979). Disorders in somesthesis following lesions of parietal lobe. J. Neurophysiol. 42, 400–419. 10.1152/jn.1979.42.2.400 106093

[B60] Lauritzen M. Mathiesen C. Schaefer K. Thomsen K. J. (2012). Neuronal inhibition and excitation, and the dichotomic control of brain hemodynamic and oxygen responses. NeuroImage 62, 1040–1050. 10.1016/j.neuroimage.2012.01.040 22261372

[B61] Lederman S. J. Klatzky R. L. (2009). Haptic perception: a tutorial. Atten. Percept. Psychophys. 71, 1439–1459. 10.3758/app.71.7.1439 19801605

[B62] Levy L. M. Ziemann U. Chen R. Cohen L. G. (2002). Rapid modulation of GABA in sensorimotor cortex induced by acute deafferentation. Ann. Neurol. 52, 755–761. 10.1002/ana.10372 12447929

[B63] Lipton M. L. Liszewski M. C. O’Connell M. N. Mills A. Smiley J. F. Branch C. A.. (2010). Interactions within the hand representation in primary somatosensory cortex of primates. J. Neurosci. 30, 15895–15903. 10.1523/JNEUROSCI.4765-09.2010 21106828 PMC3073563

[B64] Liss A. G. af Ekenstam F. W. Wiberg M. (1996). Loss of neurons in the dorsal root ganglia after transection of a peripheral sensory nerve. An anatomical study in monkeys. Scand. J. Plast. Reconstr. Surg. Hand Surg. 30, 1–6. 10.1016/s0278-2391(96)90511-x 8711436

[B65] Lofvenberg J. Johansson R. S. (1984). Regional differences and interindividual variability in sensitivity to vibration in the glabrous skin of the human hand. Brain Res. 301, 65–72. 10.1016/0006-8993(84)90403-7 6733489

[B66] Logothetis N. K. (2008). What we can do and what we cannot do with fMRI. Nature 453, 869–878. 10.1038/nature06976 18548064

[B67] Lundborg G. Rosén B. (2007). Hand function after nerve repair. Acta Physiol. 189, 207–217. 10.1111/j.1748-1716.2006.01653.x 17250571

[B68] Mackel R. (1985). Human cutaneous mechanoreceptors during regeneration: physiology and interpretation. Ann. Neurol. 18, 165–172. 10.1002/ana.410180202 4037758

[B69] Mackel R. Brink E. E. Wittkowsky G. (1983). Transitional properties of afferents reinnervating mechanoreceptors in the human glabrous skin. Brain Res. 276, 339–343. 10.1016/0006-8993(83)90743-6 6627016

[B70] Marik S. A. Yamahachi H. McManus J. N. Szabo G. Gilbert C. D. (2010). Axonal dynamics of excitatory and inhibitory neurons in somatosensory cortex. PLoS Biol. 8:e1000395. 10.1371/journal.pbio.1000395 20563307 PMC2885981

[B71] Martuzzi R. van der Zwaag W. Farthouat J. Gruetter R. Blanke O. (2014). Human finger somatotopy in areas 3b, 1, and 2: a 7T fMRI study using a natural stimulus. Hum. Brain Mapp. 35, 213–226. 10.1002/hbm.22172 22965769 PMC6869627

[B72] Merzenich M. M. Jenkins W. M. (1993). Reorganization of cortical representations of the hand following alterations of skin inputs induced by nerve injury, skin island transfers and experience. J. Hand. Ther. 6, 89–104. 10.1016/s0894-1130(12)80290-0 8393727

[B73] Mogilner A. Grossman J. A. Ribary U. Joliot M. Volkmann J. Rapaport D.. (1993). Somatosensory cortical plasticity in adult humans revealed by magnetoencephalography. Proc. Natl. Acad. Sci. U S A 90, 3593–3597. 10.1073/pnas.90.8.3593 8386377 PMC46347

[B74] Mowery T. M. Sarin R. M. Kostylev P. V. Garraghty P. E. (2015). Differences in AMPA and GABA_A/B_ receptor subunit expression between the chronically reorganized cortex and brainstem of adult squirrel monkeys. Brain Res. 1611, 44–55. 10.1016/j.brainres.2015.03.010 25791620 PMC4441862

[B75] Nelson A. J. Staines W. R. Graham S. J. McIlroy W. E. (2004). Activation in SI and SII: the influence of vibrotactile amplitude during passive and task-relevant stimulation. Cogn. Brain Res. 19, 174–184. 10.1016/j.cogbrainres.2003.11.013 15019713

[B76] Nordmark P. F. Ljungberg C. Johansson R. S. (2018). Structural changes in hand related cortical areas after median nerve injury and repair. Sci. Rep. 8:4485. 10.1038/s41598-018-22792-x 29540748 PMC5852239

[B77] Nordmark P. F. Pruszynski J. A. Johansson R. S. (2012). BOLD responses to tactile stimuli in visual and auditory cortex depend on the frequency content of stimulation. J. Cogn. Neurosci. 24, 2120–2134. 10.1162/jocn_a_00261 22721377

[B78] Nudo R. J. Plautz E. J. Milliken G. W. (1997). Adaptive plasticity in primate motor cortex as a consequence of behavioral experience and neuronal injury. Semin. Neurosci. 9, 13–23. 10.1006/smns.1997.0102

[B79] Paul R. L. Goodman H. Merzenich M. (1972). Alterations in mechanoreceptor input to Brodmann’s areas 1 and 3 of the postcentral hand area of Macaca mulatta after nerve section and regeneration. Brain Res. 39, 1–19. 10.1016/0006-8993(72)90782-2 4623626

[B80] Pederson W. C. (2014). Median nerve injury and repair. J. Hand. Surg. Am. 39, 1216–1222. 10.1016/j.jhsa.2014.01.025 24862118

[B81] Phillips J. R. Johansson R. S. Johnson K. O. (1992). Responses of human mechanoreceptive afferents to embossed dot arrays scanned across fingerpad skin. J. Neurosci. 12, 827–839. 10.1523/JNEUROSCI.12-03-00827.1992 1545242 PMC6576043

[B82] Pons T. P. Garraghty P. E. Ommaya A. K. Kaas J. H. Taub E. Mishkin M. (1991). Massive cortical reorganization after sensory deafferentation in adult macaques. Science 252, 1857–1860. 10.1126/science.1843843 1843843

[B83] Puckett A. M. Bollmann S. Barth M. Cunnington R. (2017). Measuring the effects of attention to individual fingertips in somatosensory cortex using ultra-high field (7T) fMRI. NeuroImage 161, 179–187. 10.1016/j.neuroimage.2017.08.014 28801252

[B84] Puts N. A. Edden R. A. (2012). *In vivo* magnetic resonance spectroscopy of GABA: a methodological review. Prog. Nucl. Magn. Reson. Spectrosc. 60, 29–41. 10.1016/j.pnmrs.2011.06.001 22293397 PMC3383792

[B85] Reed J. L. Qi H. X. Kaas J. H. (2011). Spatiotemporal properties of neuron response suppression in owl monkey primary somatosensory cortex when stimuli are presented to both hands. J. Neurosci. 31, 3589–3601. 10.1523/JNEUROSCI.4310-10.2011 21389215 PMC3063385

[B86] Reed J. L. Qi H. X. Zhou Z. Bernard M. R. Burish M. J. Bonds A. B.. (2010). Response properties of neurons in primary somatosensory cortex of owl monkeys reflect widespread spatiotemporal integration. J. Neurophysiol. 103, 2139–2157. 10.1152/jn.00709.2009 20164400 PMC2853283

[B7101] Riener R. Villgrattner T. Kleiser R. Nef T. Kollias S. (2005). fMRI-compatible electromagnetic haptic interface. Conf. Proc. IEEE Eng. Med. Biol. Soc. 7, 7024–7027. 10.1109/IEMBS.2005.1616123 17281892

[B87] Romo R. Lemus L. de Lafuente V. (2012). Sense, memory, and decision-making in the somatosensory cortical network. Curr. Opin. Neurobiol. 22, 914–919. 10.1016/j.conb.2012.08.002 22939031

[B88] Rosén B. (1996). Recovery of sensory and motor function after nerve repair. A rationale for evaluation. J. Hand. Ther. 9, 315–327. 10.1016/s0894-1130(96)80037-8 8994006

[B89] Ruben J. Krause T. Taskin B. Blankenburg F. Moosmann M. Villringer A. (2006). Sub-area-specific Suppressive Interaction in the BOLD responses to simultaneous finger stimulation in human primary somatosensory cortex: evidence for increasing rostral-to-caudal convergence. Cereb. Cortex 16, 819–826. 10.1093/cercor/bhj025 16162856

[B90] Sakai K. (2008). Task set and prefrontal cortex. Annu. Rev. Neurosci. 31, 219–245. 10.1146/annurev.neuro.31.060407.125642 18558854

[B91] Sammons R. P. Keck T. (2015). Adult plasticity and cortical reorganization after peripheral lesions. Curr. Opin. Neurobiol. 35, 136–141. 10.1016/j.conb.2015.08.004 26313527

[B92] Sanes J. N. Suner S. Donoghue J. P. (1990). Dynamic organization of primary motor cortex output to target muscles in adult rats. I. Long-term patterns of reorganization following motor or mixed peripheral nerve lesions. Exp. Brain Res. 79, 479–491. 10.1007/bf00229318 2340868

[B93] Seddon H. J. Medawar P. B. Smith H. (1943). Rate of regeneration of peripheral nerves in man. J. Physiol. 102, 191–215. 10.1113/jphysiol.1943.sp004027 16991601 PMC1393392

[B94] Seeley W. W. Menon V. Schatzberg A. F. Keller J. Glover G. H. Kenna H.. (2007). Dissociable intrinsic connectivity networks for salience processing and executive control. J. Neurosci. 27, 2349–2356. 10.1523/JNEUROSCI.5587-06.2007 17329432 PMC2680293

[B95] Shmuel A. Augath M. Oeltermann A. Logothetis N. K. (2006). Negative functional MRI response correlates with decreases in neuronal activity in monkey visual area V1. Nat. Neurosci. 9, 569–577. 10.1038/nn1675 16547508

[B96] Siedentopf C. M. Heubach K. Ischebeck A. Gallasch E. Fend M. Mottaghy F. M.. (2008). Variability of BOLD response evoked by foot vibrotactile stimulation: influence of vibration amplitude and stimulus waveform. NeuroImage 41, 504–510. 10.1016/j.neuroimage.2008.02.049 18424181

[B97] Song S. Abbott L. F. (2001). Cortical development and remapping through spike timing-dependent plasticity. Neuron 32, 339–350. 10.1016/s0896-6273(01)00451-2 11684002

[B98] Staines W. R. Graham S. J. Black S. E. McIlroy W. E. (2002). Task-relevant modulation of contralateral and ipsilateral primary somatosensory cortex and the role of a prefrontal-cortical sensory gating system. NeuroImage 15, 190–199. 10.1006/nimg.2001.0953 11771988

[B99] Sunderland S. (1947). Rate of regeneration in human peripheral nerves; analysis of the interval between injury and onset of recovery. Arch. Neurol. Psychiatry 58, 251–295. 10.1001/archneurpsyc.1947.02300320002001 20265595

[B100] Takesian A. E. Bogart L. J. Lichtman J. W. Hensch T. K. (2018). Inhibitory circuit gating of auditory critical-period plasticity. Nat. Neurosci. 21, 218–227. 10.1038/s41593-017-0064-2 29358666 PMC5978727

[B101] Tal Z. Geva R. Amedi A. (2017). Positive and negative somatotopic BOLD responses in contralateral versus ipsilateral penfield homunculus. Cereb. Cortex 27, 962–980. 10.1093/cercor/bhx024 28168279 PMC6093432

[B102] Talairach J. Tournoux P. (1988). Co-Planar Stereotaxic Atlas of the Human Brain. 3-Dimensional Proportional System: An Approach to Cerebral Imaging. New York, NY: Thieme.

[B103] Talbot W. H. Darian-Smith I. Kornhuber H. H. Mountcastle V. B. (1968). The sense of flutter-vibration: comparison of the human capacity with response patterns of mechanoreceptive afferents from the monkey hand. J. Neurophysiol. 31, 301–334. 10.1152/jn.1968.31.2.301 4972033

[B104] Tamè L. Braun C. Holmes N. P. Farnè A. Pavani F. (2016). Bilateral representations of touch in the primary somatosensory cortex. Cogn. Neuropsychol. 33, 48–66. 10.1080/02643294.2016.1159547 27314449

[B105] Taylor K. S. Anastakis D. J. Davis K. D. (2009). Cutting your nerve changes your brain. Brain 132, 3122–3133. 10.1093/brain/awp231 19737843

[B106] Thakur P. H. Fitzgerald P. J. Hsiao S. S. (2012). Second-order receptive fields reveal multidigit interactions in area 3b of the macaque monkey. J. Neurophysiol. 108, 243–262. 10.1152/jn.01022.2010 22457468 PMC3434610

[B107] Tommerdahl M. Simons S. B. Chiu J. S. Favorov O. Whitsel B. L. (2006). Ipsilateral input modifies the primary somatosensory cortex response to contralateral skin flutter. J. Neurosci. 26, 5970–5977. 10.1523/JNEUROSCI.5270-05.2006 16738239 PMC6675239

[B108] Trulsson M. Francis S. T. Kelly E. F. Westling G. Bowtell R. McGlone F. (2001). Cortical responses to single mechanoreceptive afferent microstimulation revealed with fMRI. NeuroImage 13, 613–622. 10.1006/nimg.2000.0723 11305890

[B109] Turrigiano G. (2012). Homeostatic synaptic plasticity: local and global mechanisms for stabilizing neuronal function. Cold Spring Harb. Perspect. Biol. 4:a005736. 10.1101/cshperspect.a005736 22086977 PMC3249629

[B110] Tzourío-Mazoyer N. Landeau B. Papathanassiou D. Crivello F. Etard O. Delcroix N.. (2002). Automated anatomical labeling of activations in SPM using a macroscopic anatomical parcellation of the MNI MRI single-subject brain. NeuroImage 15, 273–289. 10.1006/nimg.2001.0978 11771995

[B111] Wall J. T. Kaas J. H. Sur M. Nelson R. J. Felleman D. J. Merzenich M. M. (1986). Functional reorganization in somatosensory cortical areas 3b and 1 of adult monkeys after median nerve repair: possible relationships to sensory recovery in humans. J. Neurosci. 6, 218–233. 10.1523/JNEUROSCI.06-01-00218.1986 3944620 PMC6568627

[B112] van Westen D. Fransson P. Olsrud J. Rosén B. Lundborg G. Larsson E. M. (2004). Fingersomatotopy in area 3b: an fMRI-study. BMC Neurosci. 5:28. 10.1186/1471-2202-5-28 15320953 PMC517711

[B113] Worsley K. J. Andermann M. Koulis T. MacDonald D. Evans A. C. (1999). Detecting changes in nonisotropic images. Hum. Brain Mapp. 8, 98–101. 10.1002/(sici)1097-0193(1999)8:2/3<98::aid-hbm5>3.0.co;2-f 10524599 PMC6873343

[B114] Worsley K. J. Liao C. H. Aston J. Petre V. Duncan G. H. Morales F.. (2002). A general statistical analysis for fMRI data. NeuroImage 15, 1–15. 10.1006/nimg.2001.0933 11771969

[B115] Zhou X. Panizzutti R. de Villers-Sidani E. Madeira C. Merzenich M. M. (2011). Natural restoration of critical period plasticity in the juvenile and adult primary auditory cortex. J. Neurosci. 31, 5625–5634. 10.1523/JNEUROSCI.6470-10.2011 21490203 PMC3758576

